# Synergistic Ciprofloxacin-RWn Peptide Therapy Overcomes Drug Resistance in Gram-Negative Bacteria

**DOI:** 10.1021/acsomega.5c02285

**Published:** 2025-06-30

**Authors:** Bashiyar Almarwani, Nsoki Phambu, Anderson Sunda-Meya

**Affiliations:** † Department of Biology, 5717 Tennessee State University, Nashville, Tennessee 37209, United States; ‡ Department of Chemistry, Tennessee State University, Nashville, Tennessee 37209, United States; § Department of Physics, 5785 Xavier University of Louisiana, New Orleans, Louisiana 70125, United States

## Abstract

The rise of multidrug-resistant (MDR) Gram-negative bacteria necessitates novel therapeutic approaches. This study demonstrates the *in vitro* synergistic activity of ciprofloxacin (CIP), a fluoroquinolone antibiotic, combined with short arginine-tryptophan-rich antimicrobial peptides (AMPs), RWn (*n* = 4, 6, 8), against *Escherichia coli* (ATCC 25922), *Klebsiella pneumoniae* (ATCC 700603), *Pseudomonas aeruginosa* (ATCC 27853), and *Acinetobacter baumannii* (ATCC 19606). While CIP alone showed limited efficacy against *K. pneumoniae* and *A. baumannii* (MICs > 32 μg/mL), coadministration with RWn peptides significantly reduced CIP minimum inhibitory concentrations (MICs) against all tested pathogens. The combination of CIP and RW4, the shortest peptide, exhibited the most potent synergy. Specifically, the MIC of CIP against *K. pneumoniae* decreased from 32.00 ± 0 to 3.00 ± 1.00 μg/mL with RW4, a >10-fold reduction. Fractional inhibitory concentration index (FICI) values were ≤0.5 for the CIP-RW4 combination against all strains, confirming synergy. Proline incorporation into RWn generally reduced this synergistic effect. Importantly, the combinations showed no cytotoxicity against human embryonic kidney (HEK293) or human umbilical vein endothelial (HUVEC) cells at effective concentrations (MICs >32 μg/mL). These results indicate that RWn AMPs, particularly RW4, can restore the effectiveness of CIP against MDR Gram-negative bacteria, providing a promising therapeutic strategy to combat antibiotic resistance. The MICs were obtained following the broth microdilution method.

## Graphical abstract

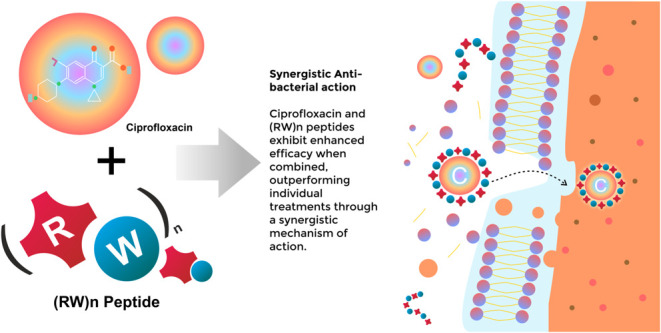

## Introduction

The rapid emergence of multidrug-resistant (MDR) Gram-negative bacteria presents a critical global health challenge, leading to increased morbidity, mortality, and healthcare costs.^1^ These pathogens are responsible for life-threatening infections, particularly in immunocompromised individuals and hospitalized patients, where they contribute significantly to the global burden of antimicrobial resistance (AMR).^2^ Key pathogens of concern include *Escherichia coli* (*Ec*), *Klebsiella pneumoniae* (*Kp*), *Pseudomonas aeruginosa* (*Pa*), and *Acinetobacter baumannii* (*Ab*), which are frequently implicated in hospital-acquired infections such as pneumonia, bloodstream infections, and urinary tract infections.^3,4^ The difficulty in treating these infections is largely attributed to the intrinsic and acquired resistance mechanisms of Gram-negative bacteria, which limit antibiotic efficacy.^5,6^

The outer membrane of Gram-negative bacteria serves as a selective permeability barrier, reducing drug penetration and contributing to antibiotic resistance.^7^ Additionally, resistance mechanisms such as efflux pump overexpression, enzymatic degradation, and target site modifications have led to widespread resistance against multiple antibiotic classes, including carbapenems, cephalosporins, and fluoroquinolones.^8,9^ Of particular concern are carbapenem-resistant enterobacteriaceae (CRE) and extensively drug-resistant (XDR) *Pa*and *Ab*, which have left clinicians with limited therapeutic options.^10,11^

Given the diminishing efficacy of conventional antibiotics and the slow pace of new drug development, novel therapeutic strategies are urgently needed.^12,13^ One promising approach is the combination of antimicrobial peptides (AMPs) with conventional antibiotics to enhance bacterial susceptibility and combat resistance mechanisms.^14,15^ AMPs, which are short, cationic peptides with broad-spectrum antimicrobial activity, disrupt bacterial membranes and exhibit lower resistance development potential compared to traditional antibiotics.^16,17^ Their synergy with conventional antibiotics can potentiate antibacterial effects, reduce required antibiotic doses, and counteract resistance mechanisms, making them viable candidates for combination therapy.^18,19^

Ciprofloxacin (CIP), a fluoroquinolone antibiotic, exerts its bactericidal activity by inhibiting bacterial DNA gyrase and topoisomerase IV, thereby interfering with DNA replication and transcription.^20^ However, its clinical utility has been undermined by resistance mechanisms such as target site mutations, increased efflux pump activity, and plasmid-mediated quinolone resistance.^21–23^ Combining CIP with AMPs targeting the bacterial membrane represents a viable strategy to restore its efficacy and mitigate resistance, as AMPs may enhance drug uptake or disrupt bacterial defense mechanisms.^24,25^

This study investigates the *in vitro* synergistic activity of CIP combined with a series of synthetic AMPs, designated RWn (where *n* = 4, 6, and 8, and “*R*” and “*W*” represent arginine and tryptophan residues, respectively), against MDR Ec, Kp, Pa, and Ab. We hypothesized that RWn peptides, particularly shorter variants, would enhance CIP’s antibacterial efficacy, lower minimum inhibitory concentrations (MICs), and circumvent resistance mechanisms.

## Materials and Methods

### Peptide Synthesis and Characterization

C-terminally amidated RWn peptides (*n* = 4, 6, 8) and their proline-substituted variants (RW4P, RW6P, RW8P, RW6–2P) were synthesized by GenScript (Piscataway, NJ) using standard solid-phase peptide synthesis (SPPS) with Fmoc (9-fluorenylmethyloxycarbonyl) chemistry.^26,27^ The specific peptides synthesized for this study, including their sequences and C-terminal modifications, are listed in Table S1 (Supporting Information). All peptides were supplied as white lyophilized powders (TFA salts).

Peptide identity and molecular weight were confirmed by matrix-assisted laser desorption-ionization time-of-flight mass spectrometry (MALDI-TOF MS) or electrospray ionization mass spectrometry (ESI-MS). Observed molecular weights were consistent with theoretical values for all peptides (Table S1, Supporting Information). Peptide purity was determined by analytical reversed-phase high-performance liquid chromatography (RP-HPLC) and confirmed to be ≥95% for all batches used (RW4:98.4%; RW4P: 96.2%; RW6:96.9%; RW6P: 96.7%; RW6–2P: 95.2%; RW8:95.8%; RW8P: 96.2%). Representative MS spectra and HPLC chromatograms for each peptide are provided in the Supporting Information (Figures S1–S14). Peptides were stored as lyophilized powders at −20 °C as recommended by the manufacturer.

### Compound Preparation and Solubility

Ciprofloxacin (CIP; Sigma-Aldrich, St. Louis, MO) and synthesized peptides were dissolved to prepare stock solutions. Based on solubility tests (Supporting Information, Solubility Reports), peptides were primarily dissolved in dimethyl sulfoxide (DMSO; analytical grade) to create high-concentration stock solutions (*e.g*., 1–10 mg/mL depending on the peptide). Notably, RW8P exhibited poor solubility in water, Dulbecco’s phosphate-buffered saline (DPBS), and DMSO, requiring formic acid for initial dissolution (≤5 mg/mL); care was taken to neutralize or highly dilute the formic acid stock before use in assays. Stock solutions were aliquoted and stored at −80 °C until use. On the day of the assay, stock solutions were thawed and further diluted in sterile water or the appropriate assay medium (*e.g*., CAMHB) to achieve the desired concentrations. The final DMSO concentration in all assay wells was maintained at ≤0.5% (v/v), a level previously shown to have no significant effect on bacterial growth. Given that peptides containing tryptophan (W) can be sensitive to oxidation in DMSO, peptide solutions in DMSO were prepared fresh or used promptly after thawing from −80 °C storage.

### Bacterial Strains and Culture Conditions

The following bacterial strains were used: *Ec* (ATCC 25922), *Kp* (ATCC 700603), *Ab*(ATCC 19606), and *Pa* (ATCC 27853). Bacteria were cultured in cation-adjusted Mueller-Hinton broth (CAMHB; Becton, Dickinson and Company, Franklin Lakes, NJ) at 37 °C with shaking.

### Minimum Inhibitory Concentration (MIC) Determination

MICs were determined in biological duplicates using the broth microdilution method according to Clinical and Laboratory Standards Institute (CLSI) guidelines.^28^ Briefly, mid-logarithmic phase bacterial cultures were diluted in CAMHB to a final concentration of 5 × 10^5^ CFU/mL. Compounds were serially diluted 2-fold in 384–well nonbinding surface (NBS) microplates (Corning, Corning, NY) to achieve a final concentration range of 0.25–32 μg/mL for both CIP and the peptides. Bacterial suspensions (50 μL) were added to each well containing the compounds (50 μL), resulting in a final volume of 100 μL. Plates were incubated at 37 °C for 18 h without shaking. Bacterial growth was assessed by measuring the optical density at 600 nm (OD_600_) using a Tecan M1000 Pro microplate reader (Tecan Group Ltd., Männedorf, Switzerland). The antimicrobial screening was performed by The Community for Antimicrobial Drug Discovery (CO-ADD).^29^

### Replicate Analysis and Reporting Format

All MIC measurements were conducted in biological duplicates. MIC values are reported as the median ± range to reflect experimental variability. This format provides a transparent summary of the central tendency and spread in exploratory synergy studies, where reproducibility is prioritized over statistical inference. When both replicates yielded identical results, MICs are expressed as median ± 0.

### Fractional Inhibitory Concentration Index (FICI) Determination

Synergy between CIP and the RWn peptides was assessed by calculating the fractional inhibitory concentration index (FICI) using the following formula:

$$FICI=\frac{{\mathrm{MIC}}_{CIP,in,combination}}{{MIC}_{CIP,alone}}+\frac{{MIC}_{RWn,in,combination}}{{MIC}_{RW,nalone}}$$

FICI values are interpreted as follows:^30^ ≤0.5, synergy; >0.5 and ≤1, additivity; >1 and ≤4, indifference; >4, antagonism. Each FICI value is reported as median ± range from two independent experiments.

### Cytotoxicity Assay

The cytotoxicity of CIP and the RWn peptides alone and in combination was evaluated against human embryonic kidney (HEK293) cells (ATCC CRL-1573) using the resazurin reduction assay. HEK293 cells were cultured in Dulbecco’s modified Eagle’s medium (DMEM; Gibco, Thermo Fisher Scientific, Waltham, MA) supplemented with 10% (v/v) fetal bovine serum (FBS; Gibco) and 1% penicillin/streptomycin (Gibco) at 37 °C in a humidified atmosphere containing 5% CO_2_. Cells were seeded in 384-well plates at a density of 5 × 10^3^ cells/well and incubated for 24 h. Compounds were then added to the wells at various concentrations. After a further 24 h incubation, resazurin (Sigma-Aldrich) was added to each well to a final concentration of 44 μM. After 4 h of incubation, fluorescence was measured at excitation/emission wavelengths of 560/590 nm using a Tecan M1000 Pro microplate reader. Cell viability was expressed as a percentage relative to that of untreated control cells. The CC_50_ (concentration causing 50% cytotoxicity) was determined by nonlinear regression analysis using GraphPad Prism software (version 9.0; GraphPad Software, San Diego, CA).

### Quality Control and Data Analysis

All assays were conducted in duplicate (*n* = 2) across separate assay plates. *Z*′-Factor analysis was used for quality control, with values >0.4 indicating acceptable assay reproducibility. Negative inhibition values indicated bacterial overgrowth relative to untreated controls.

The DMax parameter was used to determine whether compounds exhibited full or partial inhibition at tested concentrations.

## Results

### Antibacterial Activity of Ciprofloxacin and RWn Peptides Alone

The *in vitro* activities of ciprofloxacin (CIP) and the RWn peptides tested alone against the panel of Gram-negative bacteria are detailed in Table 1. CIP seems to exhibit potent activity against Pa (with a lower value of MIC ≤0.25 μg/mL) and moderate activity against Ec (with a lower value of MIC = 2 μg/mL). However, consistent with known resistance profiles, CIP demonstrated limited activity against Kp and Ab (MICs >32 μg/mL).

**Table 1:** MIC Values for Ciprofloxacin and RWn Peptides against Gram-Negative Bacteria (Median ± Range)^a^

compound(s)	*Ec*	*Kp*	*Pa*	*Ab*
CIP	3.00 ± 1.00	32.00 ± 0	16.12^b^	32.00 ± 0
RW4P	0.25 ± 0	32.00 ± 0	32.00 ± 0	32.00 ± 0
RW8P	32.00 ± 0	32.00 ± 0	32.00 ± 0	8.00 ± 0
RW6P	32.00 ± 0	32.00 ± 0	32.00 ± 0	32.00 ± 0
CIP-RW4	0.25 ± 0	3.00 ± 1.00	2.00 ± 0	8.00 ± 0
CIP-RW4P	2.50 ± 1.50	4.00 ± 0	8.80 ± 0	16.00 ± 0
CIP-RW6	0.25 ± 0	4.00 ± 0	2.00 ± 0	4.00 ± 0
CIP-RW6–2P	1.12 ± 0.88	16.16 ± 0	16.00 ± 0	20.00 ± 12.00
CIP-RW6P	2.12 ± 1.88	8.00 ± 0	8.00 ± 0	6.00 ± 2.00
CIP-RW8	1.12 ± 0.88	4.00 ± 0	3.00 ± 1.00	16.00 ± 0
CIP-RW8P	2.00 ± 0	8.00 ± 0	4.00 ± 0	16.00 ± 0
RW6–2P	16.00 ± 0	32.00 ± 0	16.12^b^	3.00 ± 1.00

a Values are expressed in μg/mL.

b Values marked with an asterisk (*e.g*., 16.12*) indicate unusually large variability between duplicate replicates. These values are shown for completeness but should be interpreted with caution as they may reflect assay variability or borderline activity.

The RWn peptides, with the exception of RW6–2P, generally showed minimal intrinsic antibacterial activity against the tested Gram-negative strains at concentrations up to 32 μg/mL (Table 1). RW6–2P, however, exhibited notable activity against *Kp* and *Ab* (with a lower value of MICs ≤0.25 μg/mL) and moderate activity against *Pa* (with a lower value of MIC = 2 μg/mL).

### Synergistic Activity of CIP and RWn Combinations

Combining CIP with the RWn peptides resulted in a significant potentiation of its activity against all four Gram-negative pathogens, particularly the strains that were less susceptible to CIP alone. As visualized in Figure 1 and detailed in Table 1, the MIC values of CIP were substantially reduced in the presence of the peptides. For instance, the MIC of CIP against the resistant Kp strain dropped from 32.00 ± 0 to 3.00 ± 1.00 μg/mL (with RW4) and 4.00 ± 0 μg/mL (with RW6 and RW8), representing >10-fold and >8-fold reductions, respectively. Similarly, against *Ab*, CIP MICs were reduced from 32.00 ± 0 to 6.00 ± 2.00 μg/mL depending on the peptide partner. Notably potent activity was also observed for CIP combined with RW6–2P, although RW6–2P itself possessed intrinsic activity.

**Figure 1:**
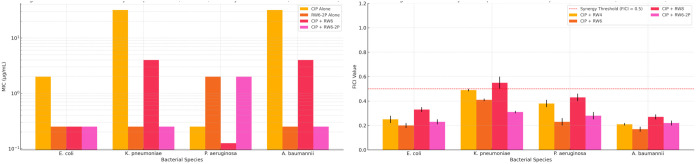
Synergistic enhancement of ciprofloxacin activity by RWn peptides. (Left) Minimum inhibitory concentrations (MICs) of ciprofloxacin (CIP) alone and RW6–2P alone and their combinations with RW6 or RW6–2P against *E. coli*, *K. pneumoniae*, *P. aeruginosa*, and *A. baumannii*. Bars represent median MIC values, and error bars represent the ranges across two independent experiments. RW6–2P demonstrated intrinsic antibacterial activity, while CIP combinations with RWn peptides substantially reduced MICs, particularly in resistant strains. (Right) Fractional inhibitory concentration index (FICI) values for CIP combined with RW4, RW6, RW8, and RW6–2P against the same bacterial panel. Bars show median FICI values in the ± range from replicate assays. The red dashed line indicates the synergy threshold (FICI ≤ 0.5). Strong synergy was observed for the CIP + RW6 and CIP + RW6–2P combinations across most strains.

### Influence of Peptide Structure on Synergistic Activity

The degree of synergy was influenced by the peptide structure. The nonprolinated peptides (RW4, RW6, RW8) generally resulted in lower CIP MICs compared to their corresponding single-proline variants (RW4P, RW6P, RW8P), particularly against *Kp* and *Ab* (Table 1). Among the nonprolinated peptides, RW6 demonstrated consistently low combination MIC values across the tested strains, although RW4 also showed strong potentiation.

### Fractional Inhibitory Concentration Index (FICI) Analysis

To quantitatively assess the interactions, FICI values were calculated for all combinations (Figure 1 and Table 2). FICI values ≤0.5 indicate synergy. Strong synergy was observed for CIP combined with RW6 against all strains, including *Kp* (FICI = 0.40). RW6–2P also demonstrated synergy against all strains. RW4 exhibited synergy against all four strains (FICI values 0.20–0.50). Combinations involving the proline variants RW4P, RW6P, and RW8P generally showed additivity (0.5 < FICI ≤ 1) or weaker synergy compared to their nonprolinated counterparts.

**Table 2:** Fractional Inhibitory Concentration Index (FICI) Values for Ciprofloxacin-RWn Peptide Combinations (Median ± Range) against Gram-Negative Bacteria^a^

combination	*Ec*	*Kp*	*Pa*	*Ab*
CIP-RW4	0.10 ± 0.06	0.19 ± 0.06	4.26 ± 8.27	0.50 ± 0.00
CIP-RW4P	12.44 ± 9.12	0.25 ± 0.00	18.75 ± 36.39	1.00 ± 0.00
CIP-RW6	0.10 ± 0.06	0.25 ± 0.00	4.26 ± 8.27	0.25 ± 0.00
CIP-RW6–2P	0.34 ± 0.43	1.01 ± 0.00	34.08 ± 66.17	1.25 ± 0.75
CIP-RW6P	0.63 ± 1.00	0.50 ± 0.00	17.04 ± 33.08	0.38 ± 0.25
CIP-RW8	0.34 ± 0.43	0.25 ± 0.00	4.32 ± 8.15	1.00 ± 0.00
CIP-RW8P	0.81 ± 0.50	0.50 ± 0.00	8.52 ± 16.54	2.50 ± 0.00

a FICI values ≤0.5 indicate synergy; 0.5 < FICI ≤ 1 indicate additive effects.

### Cytotoxicity Assessment

The potential toxicity of the peptides and their combinations with CIP was evaluated against human cell lines (HEK293 and HUVEC). As summarized in Table 3, neither the peptides alone nor their combinations with ciprofloxacin exhibited significant cytotoxicity at the highest concentrations tested (CC_50_ >32 μg/mL). Cell viability remained above 90% for all treatments. This indicates a favorable *in vitro* selectivity profile for synergistic combinations.

**Table 3:** Cytotoxicity Assessment of Ciprofloxacin and RWn Peptides in HEK293 and HUVEC Cells

compound	HEK293 (CC_50_, μg/mL)	HUVEC (CC_50_, μg/mL)
ciprofloxacin	>32	>32
RW4	>32	>32
RW6	>32	>32
CIP + RW4	>32	>32
CIP + RW6	>32	>32

## Discussion

### Synergistic Activity and Proposed Rationale

The increasing prevalence of MDR Gram-negative bacteria represents a serious clinical challenge, demanding the development of new therapeutic strategies.^1,2^ Our study demonstrates that short, arginine-tryptophan-rich peptides (RWn) can significantly potentiate the activity of CIP against clinically relevant MDR Gram-negative pathogens, including *Ec*, *Kp*, *Pa*, and *Ab*.

The observed synergy between CIP and the RWn peptides is likely due to their complementary mechanisms of action. CIP inhibits bacterial DNA gyrase and topoisomerase IV, interfering with DNA replication.^20,23^ RWn peptides, particularly those rich in arginine and tryptophan, are known to interact with and disrupt bacterial membranes.^16,17,31^ This membrane disruption likely facilitates the entry of CIP into the bacterial cell, increasing its intracellular concentration and overcoming resistance mechanisms such as efflux pumps, which are commonly observed in *Kp*and *Ab*.^8,9,32^

The finding that RW4 and RW6, two medium-length peptides, exhibited the most potent synergy with CIP is significant. Structural factors such as peptide charge and hydrophobicity likely influence this effect, as observed in previous studies where optimized amphipathic peptides demonstrated enhanced bacterial uptake.^24,33^ The reduced synergy observed with the proline-containing peptides suggests that proline-induced rigidity may hinder effective membrane interaction, as previously reported in antimicrobial peptide studies.^34^

The absence of cytotoxicity at effective antibacterial concentrations suggests that these combinations offer a favorable therapeutic window. Previous studies have shown that cationic antimicrobial peptides selectively target bacterial membranes while sparing mammalian cells, likely due to differences in lipid composition.^19,25^

### Mechanism of Synergistic Action between RWn Peptides and CIP

The observed synergy between CIP and the RWn peptides strongly suggests a multifaceted mechanism involving disruption of the Gram-negative bacterial outer membrane and potentially interference with other cellular processes.^35^ We propose the following mechanistic model, based on the known properties of arginine- and tryptophan-rich AMPs and our experimental findings:

#### Outer Membrane Permeabilization

The primary mechanism likely involves the interaction of the cationic RWn peptides with the negatively charged lipopolysaccharide (LPS) layer of the Gram-negative outer membrane^22,31,34,36,37^ . Arginine residues, with their guanidinium groups, are known to form strong electrostatic interactions with the phosphate groups of Lipid A in LPS, displacing divalent cations that stabilize the outer membrane^32,34,38^ . Tryptophan residues, with their bulky, amphipathic indole side chains, have a high affinity for the interfacial region of lipid bilayers, anchoring the peptide and promoting membrane insertion and disruption.^39–41^

This initial interaction likely leads to increased membrane permeability, either through specific mechanisms like toroidal pore formation or carpet models, or via general membrane destabilization, causing more extensive damage.^42–45^ This permeabilization allows CIP, which normally faces restricted entry across the outer membrane barrier and active removal by efflux pumps, to enter the bacterial cell more readily and reach higher intracellular concentrations.^46–48^

#### Inner Membrane Interaction (Potentially)

While the outer membrane is the primary barrier, it is possible that the RWn peptides, particularly the shorter and potentially more membrane-active RW4,^44^ also interact with and disrupt the inner (cytoplasmic) membrane, similar to other potent AMPs.^42^ This could further contribute to bacterial killing by dissipating the proton motive force and causing leakage of essential cytoplasmic contents, while also enhancing CIP’s access to its intracellular targets (DNA gyrase and topoisomerase IV).^49,50^

Furthermore, arginine-rich peptides, often termed cell-penetrating peptides (CPPs), have been shown to translocate across bacterial membranes and even interact with intracellular targets such as nucleic acids or ribosomes, although this often requires specific structural motifs not solely defined by (RW)­n repeats.^51–55^ While less likely to be the dominant mechanism of synergy here compared with gross permeabilization facilitating CIP uptake, intracellular activity cannot be entirely ruled out as a contributing factor.

#### Efflux Pump Inhibition (Potentially)

Some AMPs have been reported to inhibit efflux pumps, either directly by interacting with pump proteins or indirectly by disrupting the membrane potential required for pump activity. Efflux pumps, such as the AcrAB-TolC system, are major contributors to fluoroquinolone resistance in Gram-negative bacteria.^56,57^ Several studies have demonstrated that certain AMPs can act as efflux pump inhibitors or potentiate antibiotics by counteracting efflux.^57–59^ While we did not directly assess efflux pump inhibition in this study, it remains a possibility that the RWn peptides, through membrane disruption and potential interactions with pump components or energetics, could reduce the efflux of CIP, further increasing its intracellular concentration and contributing to the observed synergy.

#### Influence of Peptide Structure

The structure of the RWn peptides clearly influenced their synergistic potential with CIP. While potent synergy was observed with certain peptides against specific strains (*e.g*., the high FICI synergy values for RW6–2P against *Kp*), the shorter, nonprolinated peptide RW4 stood out for its consistent synergistic activity (FICI ≤0.5) across all four diverse Gram-negative species tested. This broad-spectrum potentiation, coupled with its structural simplicity as the shortest active peptide in the nonprolinated series, highlights RW4 as a promising lead scaffold from this study.

Both RW4 and RW6 demonstrated strong synergistic activity with ciprofloxacin; however, RW4 was selected as the most promising lead due to its superior safety profile. As reported in our previously published work,^60^ RW4 exhibited no detectable cytotoxicity against human embryonic kidney (HEK293) or human umbilical vein endothelial (HUVEC) cells at concentrations exceeding its antibacterial MICs (>32 μg/mL), as further confirmed in this study (Table 3).

This observation aligns with structure–activity relationships often seen in AMPs, where peptide length and conformational flexibility are crucial for optimal membrane interaction.^44,45^ Shorter peptides like RW4 may possess greater flexibility, potentially allowing for more efficient insertion into diverse bacterial membranes and the induction of disruptive curvature strain or transient pores compared to the longer RW6 and RW8 peptides.^61,62^

Conversely, the generally reduced synergy observed with the single-proline-containing variants (RW4P, RW6P, RW8P) compared to their nonprolinated counterparts supports the hypothesis that proline-induced structural rigidity negatively impacts activity. Proline residues introduce kinks into the peptide backbone, likely hindering the adoption of amphipathic secondary structures required for effective membrane perturbation.^63,64^

The high synergy observed specifically for RW6–2P (containing two prolines) against certain strains, despite the generally negative impact of single prolines, is intriguing. This suggests that its specific sequence and structure might facilitate a distinct mode of interaction or potentially engage with targets beyond simple membrane disruption, warranting further investigation. Overall, the consistent broad-spectrum synergy of the structurally simpler RW4 underscores its potential for further development as an adjuvant to CIP.

#### Synergistic Advantage

The presumed multipronged mechanism, primarily enhanced drug uptake via membrane permeabilization, potentially aided by inner membrane disruption and/or efflux inhibition, likely explains the strong synergy observed. By targeting the bacterial envelope integrity, the RWn peptides compromise the cell’s defenses, making it significantly more susceptible to the intracellular action of CIP. This multitarget approach, hitting both the barrier function and the DNA replication machinery, inherently reduces the likelihood of bacteria developing resistance compared to treatment with a single-target antibiotic.^65–68^

Despite these promising findings, our study has some limitations. It is an *in vitro* study, and further *in vivo* investigations are necessary to confirm the efficacy and safety in an animal model. Additionally, testing against a broader panel of clinical isolates with diverse resistance mechanisms strengthens the translational relevance of these findings.

## Conclusions

The global rise of multidrug-resistant (MDR) Gram-negative bacteria demands new therapeutic strategies. This study demonstrates that short, arginine-tryptophan-rich antimicrobial peptides (RWn), particularly RW4, synergistically enhance the *in vitro* antibacterial activity of CIP against clinically relevant MDR strains of *Ec*, *Kp*, *Pa*, and *Ab*. The combination of CIP and RW4 resulted in significant reductions in CIP MICs, with FICI values confirming strong synergy. While RW6–2P showed good synergy, our results highlight RW4 for its superior potentiation of CIP across all of the tested strains. The observed synergy likely stems from RWn-mediated bacterial membrane disruption, facilitating CIP entry and overcoming resistance mechanisms. Importantly, these combinations exhibited minimal cytotoxicity against mammalian cells at their effective concentrations. Although further *in vivo* studies are crucial to evaluate pharmacokinetics, efficacy, and safety, our findings suggest that RWn peptides, especially RW4, represent promising antibiotic adjuvants. These peptide-antibiotic combinations offer a potential strategy to revitalize CIP’s effectiveness and address the critical threat of MDR Gram-negative infections. Future work should focus on *in vivo* validation and optimizing the RWn peptide structure for enhanced therapeutic potential.

## Supplementary Material



## Notes

Characterization, purity, and solubility data for the RWn peptides; their synthesis; identity; and handling as described in the main manuscript (PDF)
