# Supplementary material for: Synergistic Ciprofloxacin-RWn Peptide Therapy Overcomes Drug Resistance in Gram-Negative Bacteria
Source: ACS Omega. 2025 Jun 30;10(32):35698–705. doi: 10.1021/acsomega.5c02285 (PMC12368660; doi:10.1021/acsomega.5c02285)
Supplement: Supplementary file 1 [file ao5c02285_si_001.pdf]

Synergistic Ciprofloxacin-RWn Peptide Therapy Overcomes Drug Resistance in Gram-Negative Bacteria

Bashiyar Almarwani[1], Nsoki Phambu[2], and Anderson Sunda-Meya[3*]

[1]Department of Biology, Tennessee State University, Nashville, TN 37209, USA

[2]Department of Chemistry, Tennessee State University, Nashville, TN 37209, USA

[3]Department of Physics, Xavier University of Louisiana, New Orleans, LA 70125, USA

KEYWORDS: Antimicrobial Peptides (AMPs); Ciprofloxacin; Synergy; Gram-Negative Bacteria; Antibiotic Resistance; Multidrug Resistance (MDR).

Supplementary Information

This document provides supplementary data supporting the materials, methods, and findings presented in the main manuscript. The information included details the synthesis, characterization, and solubility of the RWn peptides used in this study.

Contents:

1. **Table S1:** Summary of Synthetic RWn Peptide Characterization. This table lists the peptide identifiers, full amino acid sequences, C-terminal modifications (if any), peptide length (amino acids), theoretical molecular weight (MW), observed molecular weight as determined by mass spectrometry (MS), and purity as determined by High-Performance Liquid Chromatography (HPLC).
2. **Figures S1-S13:** Peptide Characterization Data (Mass Spectrometry and HPLC). These figures present the representative mass spectra (ESI-MS or MALDI-TOF MS) used to confirm the identity and molecular weight for all seven peptides. Analytical RP-HPLC chromatograms used to determine the purity (>95%) are provided for RW4, RW4P, RW6P, RW6-2P, RW8, and RW8P. For peptide RW6, the HPLC purity is reported from its Certificate of Analysis (see Table S1).
3. **Table S2:** Peptide Solubility Test Results. This table summarizes the observed solubility of each peptide in ultrapure water, 1x Dulbecco's Phosphate-Buffered Saline (DPBS, pH 7.1 ± 0.1), and Dimethyl Sulfoxide (DMSO) at defined concentration ranges. Solubility in formic acid is noted for RW8P. Results are defined as 'Dissolved' (≥0.1 mg/mL) or 'Undissolved' (<0.1 mg/mL), with estimated concentration ranges provided for dissolved samples based on vendor reports.

This supplementary information validates the identity, purity, and handling properties of the peptides described in the main manuscript's Materials and Methods section and supports the reproducibility of the reported biological assays.

Table S1. Characterization of synthetic RWn peptides used in this study.

Peptide ID	Sequence	C-terminal Modification	Length (AA)	Theoretical MW (Da)	Observed MW (MS, Da)[a]	Purity (HPLC, %)[b]
RW4	RWRWRWRW	None	8	1387.6	1387.2	98.4
RW4P	RWRWPRWRW	Amidation (-NH_2)	9	1483.74	1483.6	96.2
RW6	RWRWRWRWRWRW	None	12	2072.4	2071.5	96.9[c]
RW6P	RWRWRWPRWRWRW	Amidation (-NH_2)	13	2168.53	2168	96.7
RW6-2P	RWRWPRWRWPRWRW	Amidation (-NH_2)	14	2265.65	2266	95.2
RW8	RWRWRWRWRWRWRWRW	None	16	2757.19	2756.1	95.8
RW8P	RWRWRWRWPRWRWRWRW	Amidation (-NH_2)	17	2853.33	2853	96.2

Footnotes:

[a] Observed molecular weight determined by ESI-MS or MALDI-TOF MS, as reported in the corresponding MS analysis files (see Figures S1-S14 for representative spectra).

[b] Purity determined by analytical RP-HPLC at 220 nm, as reported in the corresponding HPLC analysis files (see Figures S1-S14 for representative chromatograms). All peptides met the vendor's specification of ≥95.0%.

[c] For peptide RW6, the analytical RP-HPLC chromatogram was not available for inclusion; purity is reported from the Certificate of Analysis provided by the vendor.

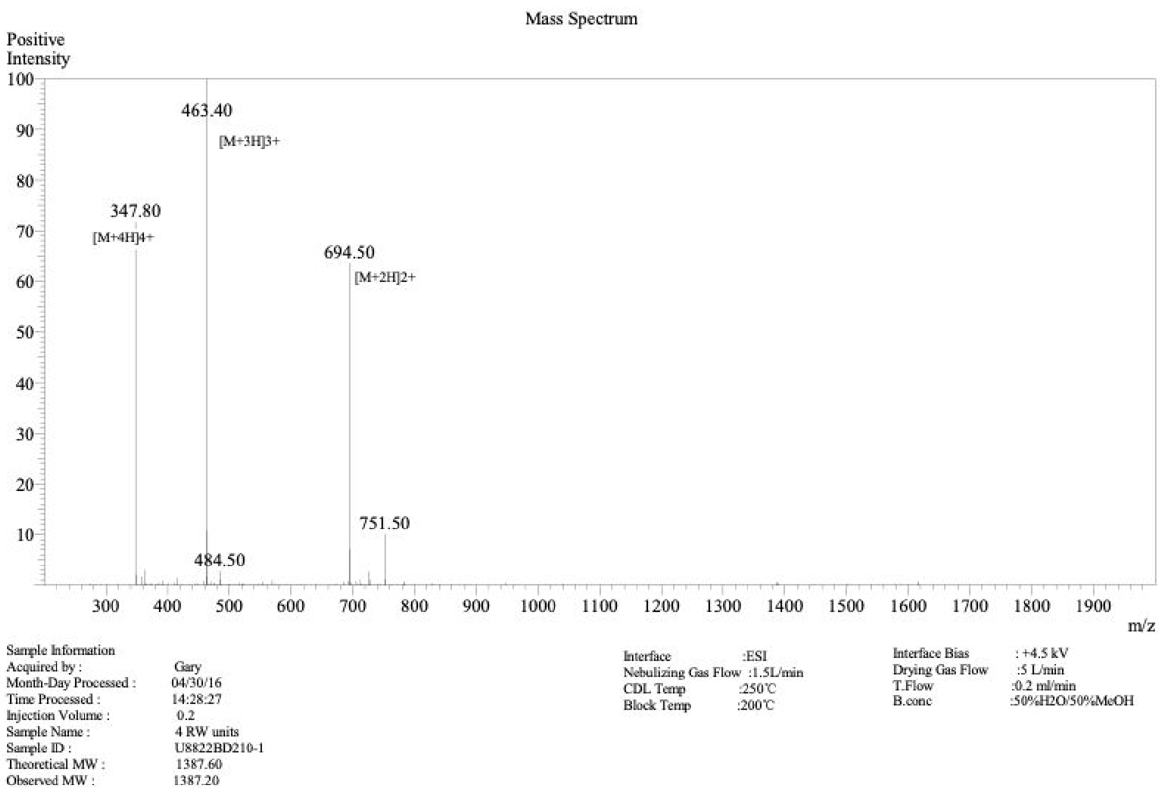

Figure S1. Mass Spectrum (ESI-MS) of RW4. Theoretical MW: 1387.60 Da. Observed MW: 1387.20 Da.

Sample Name :4 RW units
Sample ID : U8822BD210-1

Pump A : 0.065% trifluoroacetic in 100% water (v/v)
Pump B : 0.05% trifluoroacetic in 100% acetonitrile (v/v)
Total Flow:1 ml/min
Wavelength:220 nm

Time	Unit	Command	Value	Comment
0.01	Pumps	Pump A B.Conc	5	
25.00	Pumps	Pump A B.Conc	65	
25.01	Pumps	Pump A B.Conc	95	
31.00	Pumps	Pump A B.Conc	95	
31.01	Pumps	Pump A B.Conc	5	
40.00	Pumps	Pump A B.Conc	5	
40.01	Controller	Stop		

<<Column Performance>>
<Detector A>
Column : AlltimaTM C18 4.6 x 250 mm

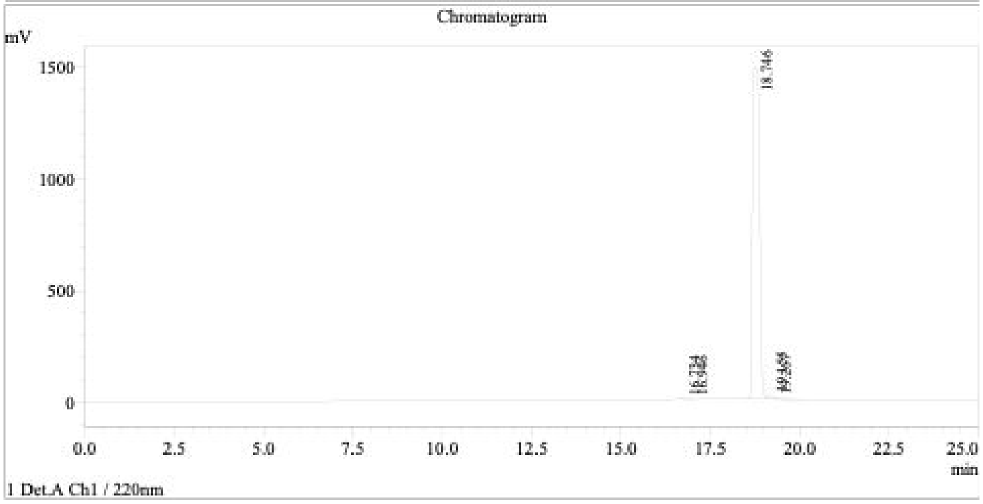

Peak Table

Detector A Ch1 220nm

Peak#	Ret. Time	Area	Height	Area %
1	16.734	15800	2451	0.075
2	16.946	15505	2702	0.074
3	18.746	20708519	1492741	98.399
4	19.155	167161	19023	0.794
5	19.267	138392	7976	0.658
Total		21045377	1524894	100.000

Figure S2. Analytical RP-HPLC Chromatogram of RW4. Purity: 98.4%. Retention time (Rt) of main peak: 18.746 min. Conditions: Alltima C18 column (4.6 x 250 mm), gradient elution, 220 nm detection.

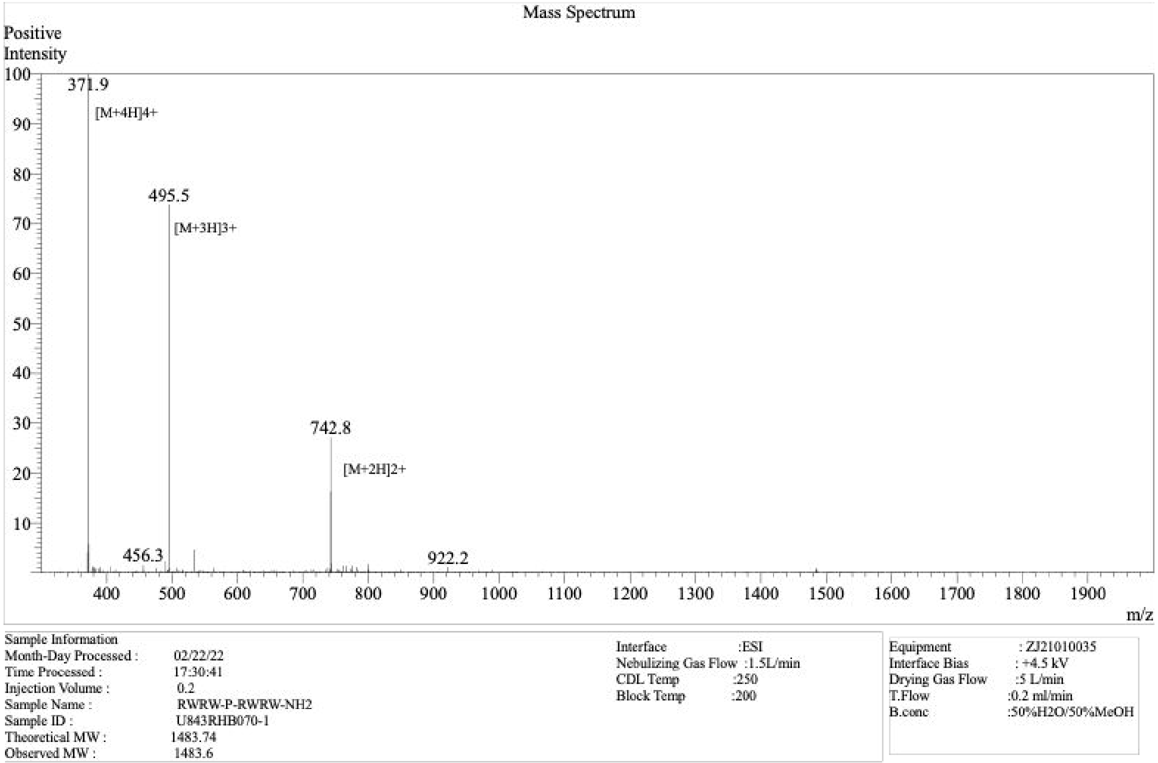

Figure S3. Mass Spectrum (ESI-MS) of RW4P. Theoretical MW: 1483.74 Da. Observed MW: 1483.6 Da.

Sample Name	:RWRW-P-RWRW-NH2
Sample ID	:U843RHB070-1
Time Processed	:21:42:47
Month-Day-Year Processed	:02/22/2022

Pump A : 0.065% trifluoroacetic in 100% water (v/v)
Pump B : 0.05% trifluoroacetic in 100% acetonitrile (v/v)
Total Flow:1 ml/min
Wavelength:220 nm
<<LC Time Program>>

Time	Module	Command	Value
0.01	Pumps	B.Conc	15
25.00	Pumps	B.Conc	75
25.01	Pumps	B.Conc	95
27.00	Pumps	B.Conc	95
27.01	Pumps	B.Conc	5
31.00	Pumps	B.Conc	5
31.01	Controller	Stop	

<<Column Performance>>
<Detector A>
Column :Inertsil ODS-3 4.6 x 250 mm
Equipment: ZJ19010324

<Chromatogram>

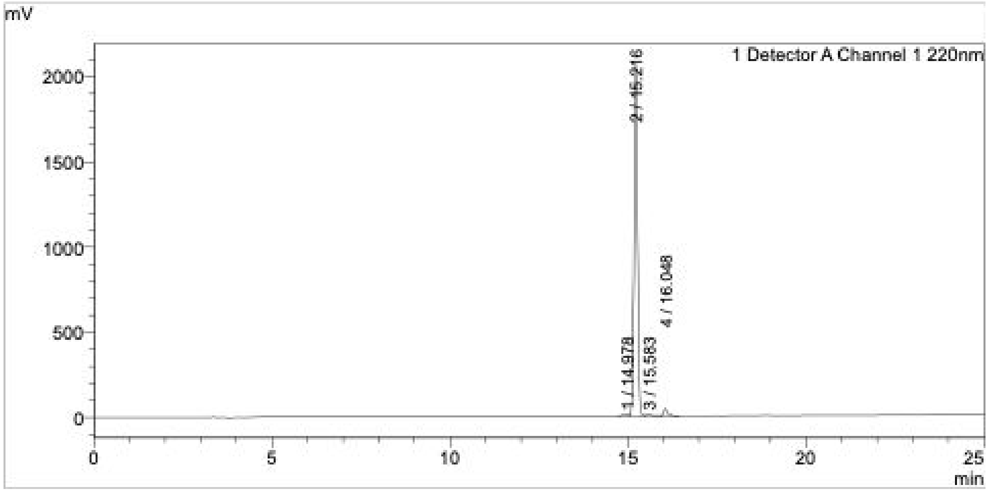

<Peak Table>

Detector A Channel 1 220nm

Peak#	Ret. Time	Area	Height	Area%
1	14.978	107478	10973	0.756
2	15.216	13677098	2064069	96.244
3	15.583	91270	8211	0.642
4	16.048	335032	43343	2.358
Total		14210877	2126596	100.000

Figure S4. Analytical RP-HPLC Chromatogram of RW4P. Purity: 96.2%. Retention time (Rt) of main peak: 15.216 min. Conditions: Inertsil ODS-3 column (4.6 x 250 mm), gradient elution, 220 nm detection.

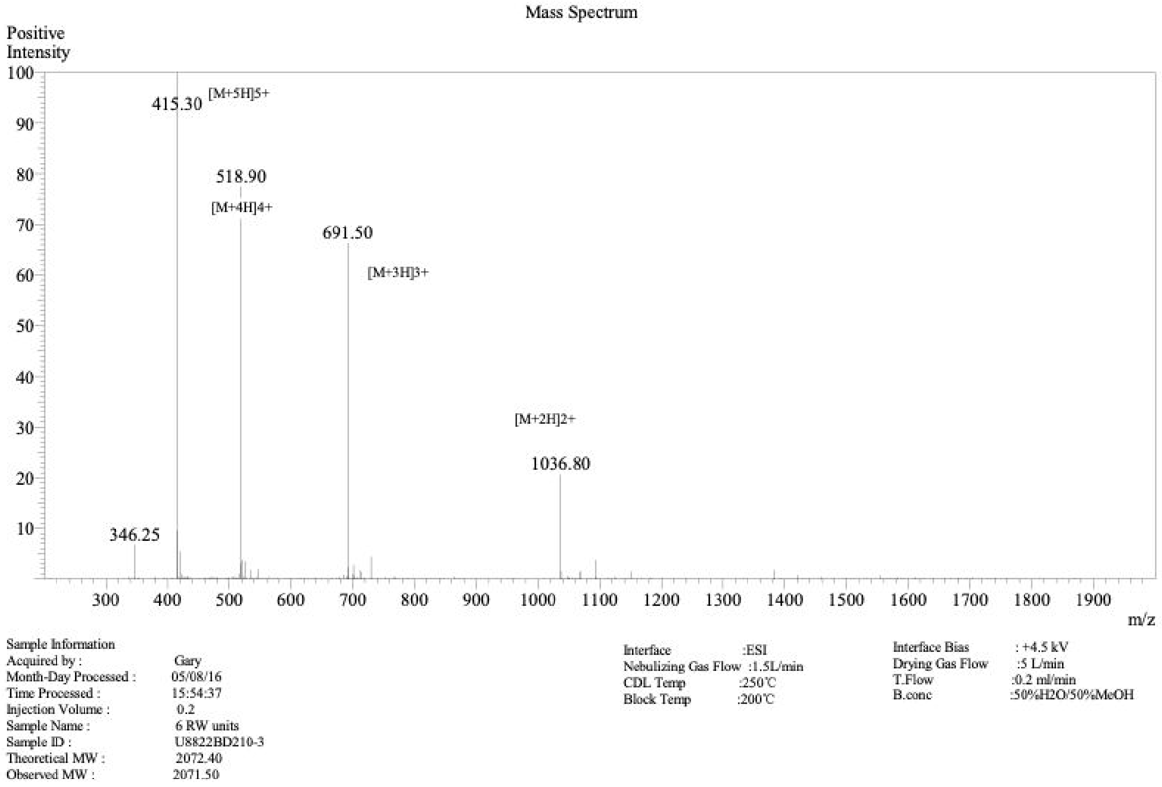

Figure S5. Mass Spectrum (ESI-MS) of RW6. Theoretical MW: 2072.40 Da. Observed MW: 2071.50 Da.

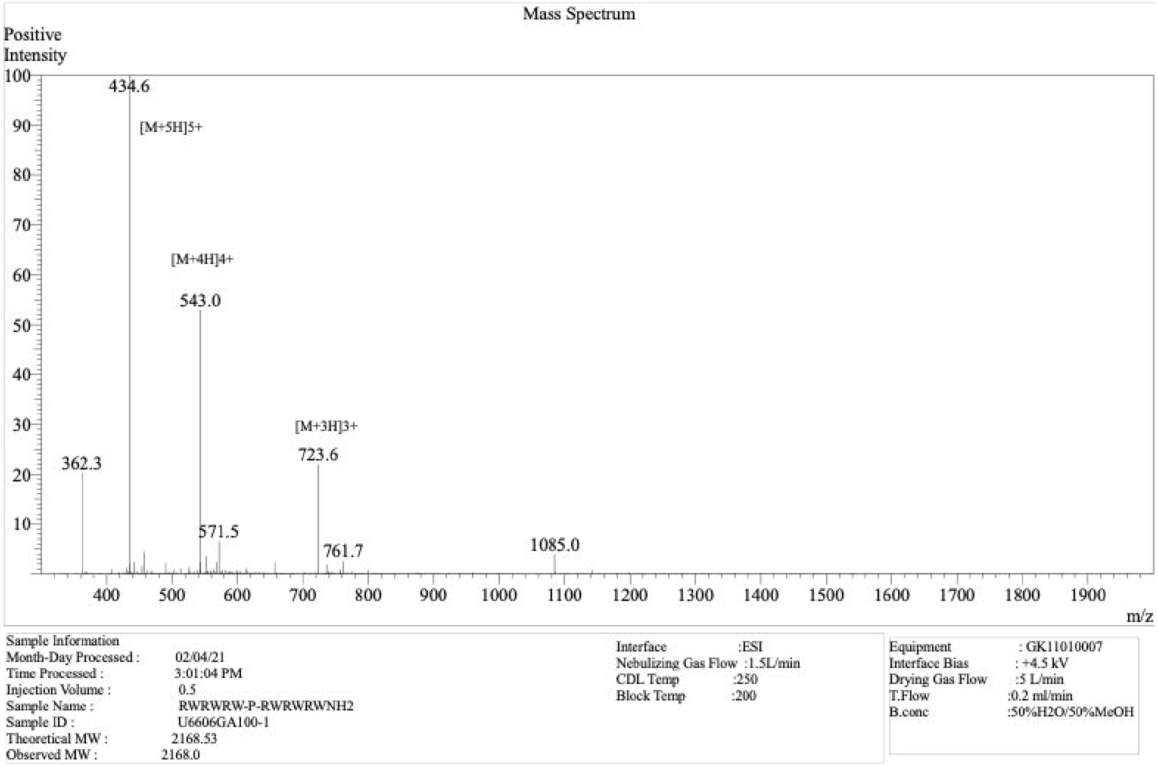

Figure S6. Mass Spectrum (ESI-MS) of RW6P. Theoretical MW: 2168.53 Da. Observed MW: 2168.0 Da.

Sample Name :RWRWRW-P-RWRWRWNH2
Sample ID :U6606GA100-1
Time Processed :14:45:13
Month-Day-Year Processed :02/04/2021

Pump A : 0.065% trifluoroacetic in 100% water (v/v)
Pump B : 0.05% trifluoroacetic in 100% acetonitrile (v/v)
Total Flow:1 ml/min
Wavelength:220 nm
<<LC Time Program>>

Time	Module	Command	Value
0.01	Pumps	B.Conc	5
25.00	Pumps	B.Conc	65
25.01	Pumps	B.Conc	95
27.00	Pumps	B.Conc	95
27.01	Pumps	B.Conc	5
35.00	Pumps	B.Conc	5
35.01	Controller	Stop	

<<Column Performance>>
<Detector A>
Column :Inertsil ODS-3 4.6 x 250 mm
Equipment: SS-CM-0310

<Chromatogram>

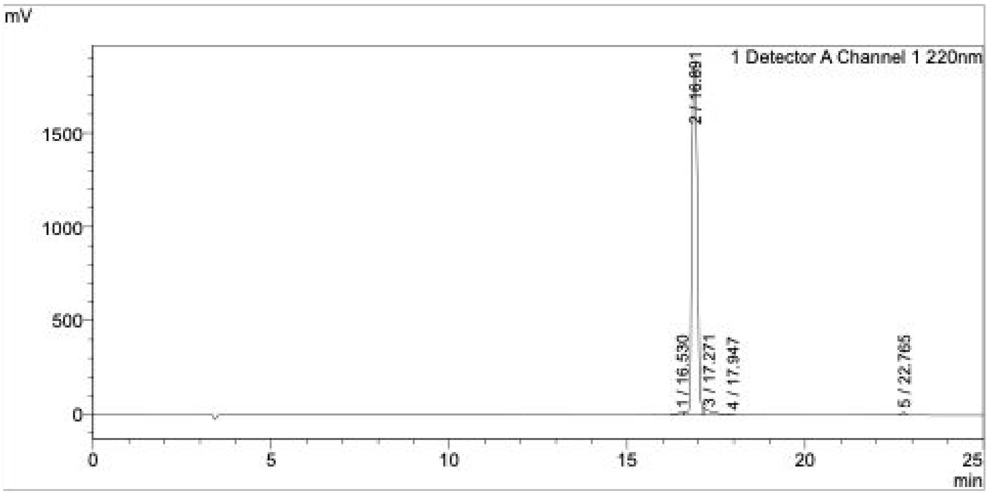

<Peak Table>

Detector A Channel 1 220nm

Peak#	Ret. Time	Area	Height	Area%
1	16.530	155971	11566	0.757
2	16.891	19913280	1859437	96.698
3	17.271	448121	19354	2.176
4	17.947	4064	683	0.020
5	22.765	71820	12074	0.349
Total		20593255	1903115	100.000

Figure S7. Analytical RP-HPLC Chromatogram of RW6P. Purity: 96.7%. Retention time (Rt) of main peak: 16.891 min. Conditions: Inertsil ODS-3 column (4.6 x 250 mm), gradient elution, 220 nm detection.

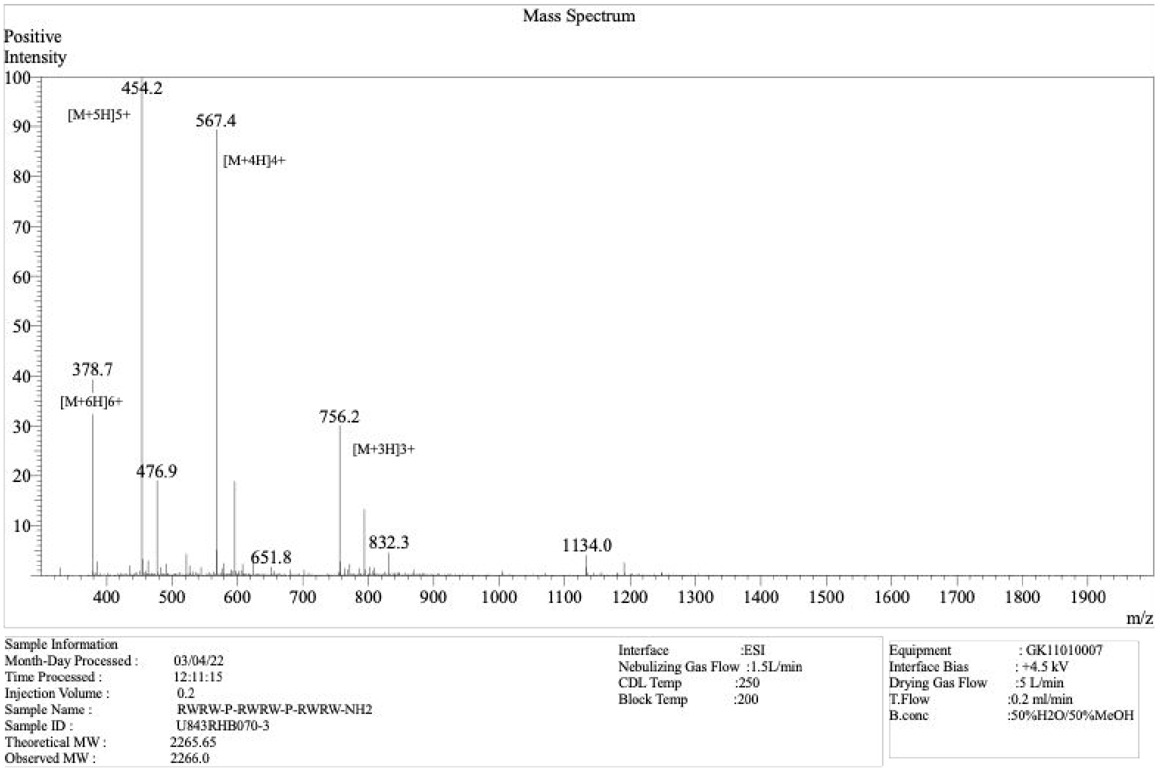

Figure S8. Mass Spectrum (ESI-MS) of RW6-2P. Theoretical MW: 2265.65 Da. Observed MW: 2266.0 Da.

Sample Name :RWRW-P-RWRW-P-RWRW-NH2
Sample ID :U843RHB070-3
Time Processed :10:10:11
Month-Day-Year Processed :03/04/2022

Pump A : 0.065% trifluoroacetic in 100% water (v/v)
Pump B : 0.05% trifluoroacetic in 100% acetonitrile (v/v)
Total Flow:1 ml/min
Wavelength:220 nm
<<LC Time Program>>

Time	Module	Command	Value
0.01	Pumps	B.Conc	5
25.00	Pumps	B.Conc	65
25.01	Pumps	B.Conc	95
30.00	Pumps	B.Conc	95
35.01	Pumps	B.Conc	30
40.00	Pumps	B.Conc	30
95.00	Controller	Stop	

<<Column Performance>>
<Detector A>
Column :Inertsil ODS-3 4.6 x 250 mm
Equipment: ZJ19010324

<Chromatogram>

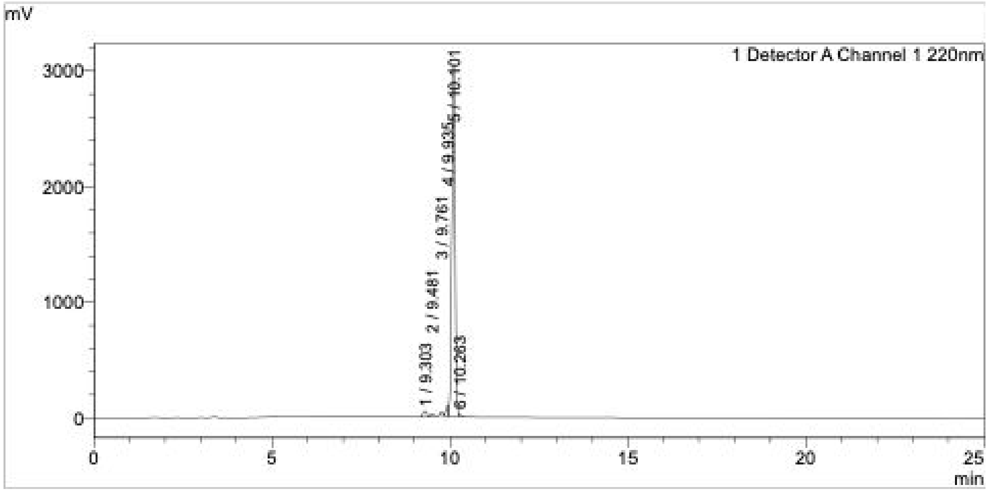

<Peak Table>

Detector A Channel 1 220nm

Peak#	Ret. Time	Area	Height	Area%
1	9.303	273584	43083	1.293
2	9.481	94420	17447	0.446
3	9.761	191631	34697	0.905
4	9.935	403558	84684	1.907
5	10.101	20142460	3046333	95.171
6	10.263	58902	16161	0.278
Total		21164556	3242405	100.000

Figure S9. Analytical RP-HPLC Chromatogram of RW6-2P. Purity: 95.2%. Retention time (Rt) of main peak: 10.101 min. Conditions: Inertsil ODS-3 column (4.6 x 250 mm), gradient elution, 220 nm detection.

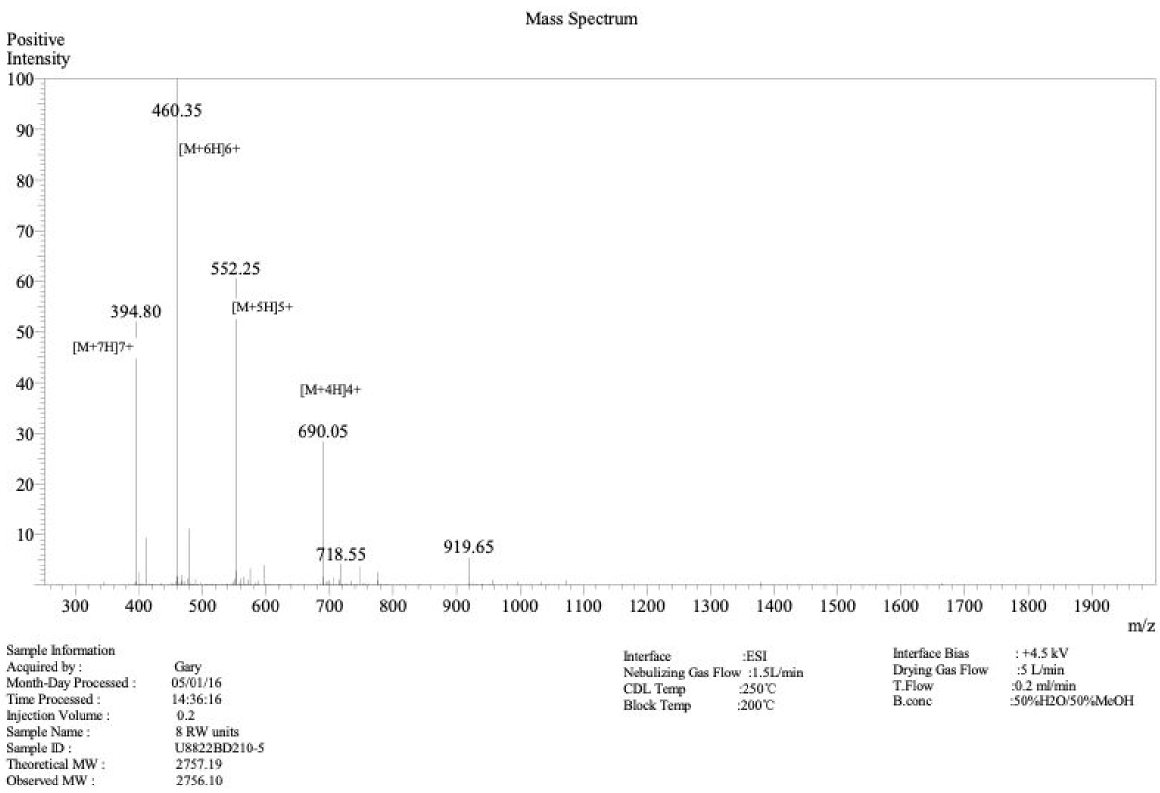

Figure S10. Mass Spectrum (ESI-MS) of RW8. Theoretical MW: 2757.19 Da. Observed MW: 2756.10 Da.

Sample Name : 8 RW units
Sample ID : U8822BD210-5

Pump A : 0.065% trifluoroacetic in 100% water (v/v)
Pump B : 0.05% trifluoroacetic in 100% acetonitrile (v/v)
Total Flow:1 ml/min
Wavelength:220 nm

Time	Unit	Command	Value	Comment
0.01	Pumps	Pump A B.Conc	5	
25.00	Pumps	Pump A B.Conc	65	
25.01	Pumps	Pump A B.Conc	95	
31.00	Pumps	Pump A B.Conc	95	
31.01	Pumps	Pump A B.Conc	5	
40.00	Pumps	Pump A B.Conc	5	
40.01	Controller	Stop		

<<Column Performance>>
<Detector A>
Column : AlltimaTM C18 4.6 x 250 mm

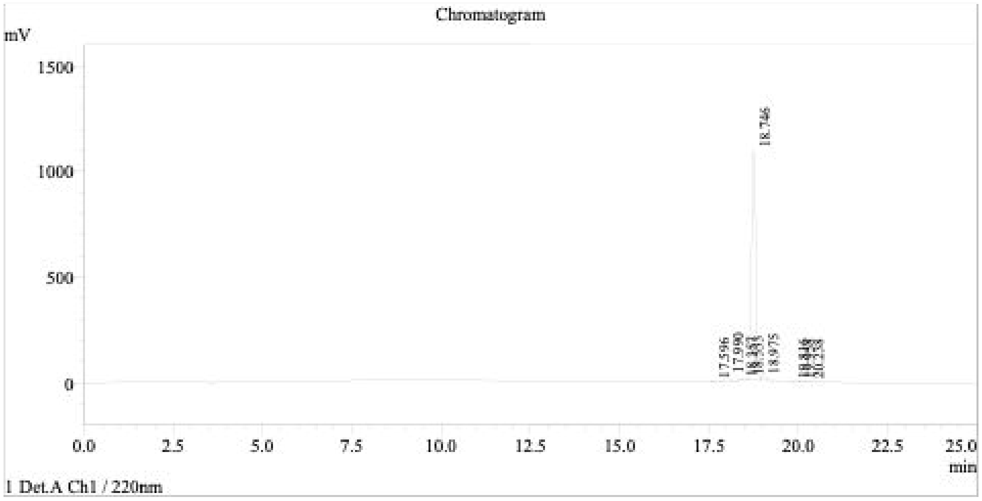

Peak Table

Detector A Ch1 220nm

Peak#	Ret. Time	Area	Height	Area %
1	17.596	12753	2462	0.134
2	17.990	198594	24358	2.088
3	18.357	21688	3804	0.228
4	18.533	9826	1515	0.103
5	18.746	9108766	1086208	95.767
6	18.975	123133	13842	1.295
7	19.816	3521	671	0.037
8	19.958	18485	2166	0.194
9	20.238	14632	2882	0.154
Total		9511397	1137908	100.000

Figure S11. Analytical RP-HPLC Chromatogram of RW8. Purity: 95.8%. Retention time (Rt) of main peak: 18.746 min. Conditions: Alltima C18 column (4.6 x 250 mm), gradient elution, 220 nm detection.

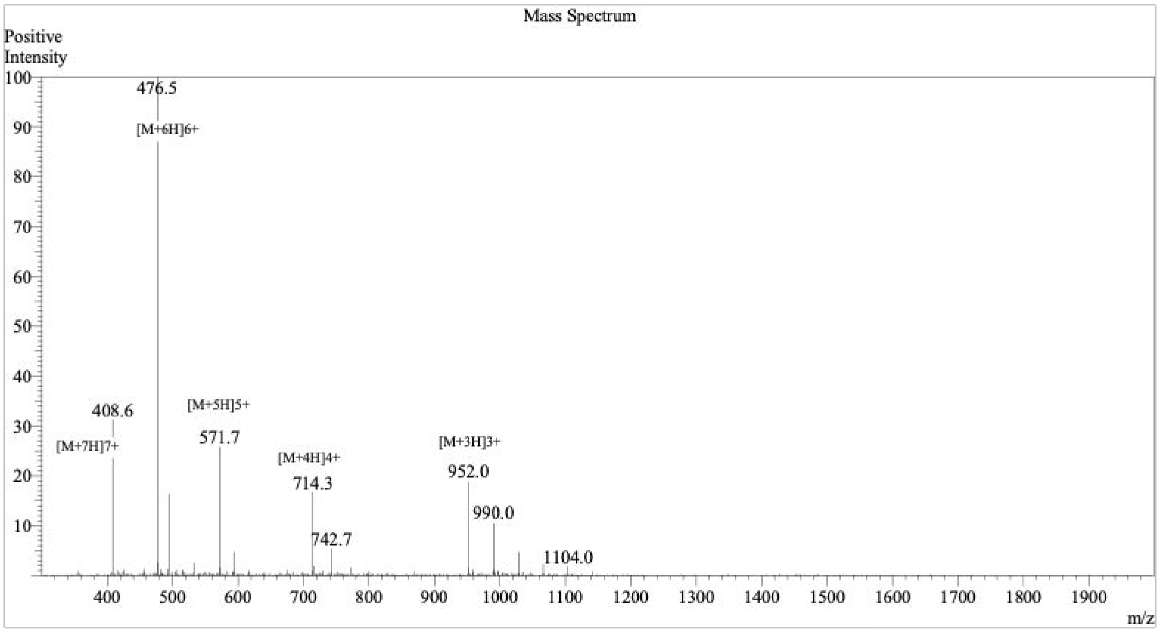

Sample Information
Month-Day Processed : 03/02/22
Time Processed : 13:54:04
Injection Volume : 0.1
Sample Name : RWRWRWRW-P-RWRWRWRW-NH2
Sample ID : U892JHB070-1
Theoretical MW : 2853.33
Observed MW : 2853.0

Interface :ESI
Nebulizing Gas Flow :1.5L/min
CDL Temp :250
Block Temp :200

Equipment : ZJ21010035
Interface Bias : +4.5 kV
Drying Gas Flow :5 L/min
T.Flow :0.2 ml/min
B.conc :50%H2O/50%MeOH

Figure S12. Mass Spectrum (ESI-MS) of RW8P. Theoretical MW: 2853.33 Da. Observed MW: 2853.0 Da.

Sample Name :RWRWRWRW-P-RWRWRWRW-NH2
Sample ID :U892JHB070-1
Time Processed :14:42:56
Month-Day-Year Processed :03/03/2022

Pump A : 0.065% trifluoroacetic in 100% water (v/v)
Pump B : 0.05% trifluoroacetic in 100% acetonitrile (v/v)
Total Flow:1 ml/min
Wavelength:220 nm
<<LC Time Program>>

Time	Module	Command	Value
0.01	Pumps	B.Conc	5
25.00	Pumps	B.Conc	65
25.01	Pumps	B.Conc	95
27.00	Pumps	B.Conc	95
27.01	Pumps	B.Conc	5
31.00	Pumps	B.Conc	5
31.00	Controller	Stop	

<<Column Performance>>
<Detector A>
Column :Inertsil ODS-3 4.6 x 250 mm
Equipment: ZJ19010324

<Chromatogram>

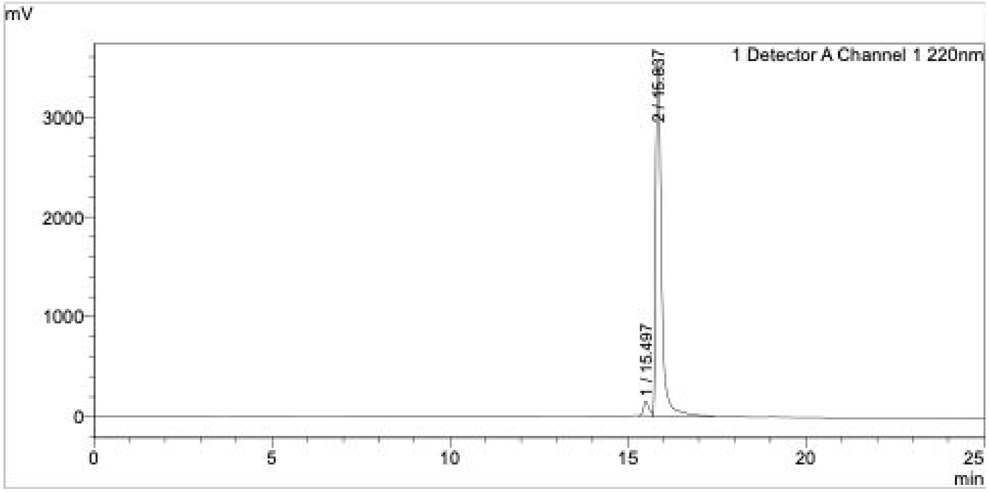

<Peak Table>

Detector A Channel 1 220nm

Peak#	Ret. Time	Area	Height	Area%
1	15.497	1543336	146459	3.780
2	15.837	39283481	3520747	96.220
Total		40826817	3667206	100.000

Figure S13. Analytical RP-HPLC Chromatogram of RW8P. Purity: 96.2%. Retention time (Rt) of main peak: 15.837 min. Conditions: Inertsil ODS-3 column (4.6 x 250 mm), gradient elution, 220 nm detection.

Table S2. Solubility Test Results for Synthetic RWn Peptides.

**Peptide ID**	**Ultrapure Water**[a]	**1x DPBS (pH 7.1 ± 0.1)**[a]	**DMSO**[a]	**Formic Acid (HCOOH)**[a]
RW4	Dissolved (≤10 mg/mL)	Dissolved (≤5 mg/mL)	Dissolved (≤15 mg/mL)	N/A
RW4P	Dissolved (≤2 mg/mL)	Dissolved (≤2 mg/mL)	Dissolved (≤10 mg/mL)	N/A
RW6	Dissolved (≤10 mg/mL)	Undissolved	Dissolved (≥10 mg/mL)	N/A
RW6P	Dissolved (≤5 mg/mL)	Undissolved	Dissolved (≥10 mg/mL)	N/A
RW6-2P	Undissolved	Undissolved	Dissolved (≤2 mg/mL)	N/A
RW8	Dissolved (≤5 mg/mL)	Undissolved	Dissolved (≤15 mg/mL)	N/A
RW8P	Undissolved	Undissolved	Undissolved	Dissolved (≤5 mg/mL)

Footnotes:

[a] Solubility determined by vendor (GenScript) based on visual inspection according to their standard protocol. "Dissolved" defined as solubility ≥0.1 mg/mL; "Undissolved" defined as solubility <0.1 mg/mL. Estimated maximum concentration (mg/mL) tested or achieved provided for dissolved samples. N/A (Not Applicable) indicates the solvent was not reported in the test for that specific peptide. Data extracted from vendor-provided Peptide Solubility Test Reports.
